# Identifying strategies to characterize the diversity of the MS population in Canada: a nominal group study

**DOI:** 10.3389/fneur.2026.1757659

**Published:** 2026-03-31

**Authors:** Ruth Ann Marrie, Afolasade Fakolade, Colleen J. Maxwell, Dalia L. Rotstein, Helen Tremlett, E. Ann Yeh, Marcia Finlayson

**Affiliations:** 1 Departments of Medicine and Community Health and Epidemiology, Faculty of Medicine, Dalhousie University, Halifax, NSW, Canada; 2 Nova Scotia Health, Halifax, NSW, Canada; 3 School of Rehabilitation Therapy, Queen’s University, Kingston, ON, Canada; 4 Schools of Pharmacy and Public Health Sciences, University of Waterloo, Waterloo, ON, Canada; 5 Department of Medicine, University of Toronto, Toronto, ON, Canada; 6 St. Michael’s Hospital, Toronto, ON, Canada; 7 Department of Medicine, The Djavad Mowafaghian Centre for Brain Health, University of British Columbia, Vancouver, BC, Canada; 8 Division of Neurology, Department of Paediatrics, The Hospital for Sick Children, University of Toronto, Toronto, ON, Canada

**Keywords:** Canada, diversity, epidemiology, multiple sclerosis, nominal group technique, social determinants of health

## Abstract

**Background:**

Diversity of the multiple sclerosis (MS) population in Canada is unknown, yet demographic and diversity-related characteristics influence health outcomes.

**Objective:**

To determine “What strategy would best address the information gaps regarding diversity of the MS population in Canada?”

**Methods:**

The virtual nominal group technique involved silent idea generation, recording and discussion of items, preliminary voting, discussion and final ranking.

**Results:**

Eight participants proposed 10 strategies; two were dropped before final voting, after which the top options (tied) were: a national standardized clinic form, novel applications of administrative data, and novel data linkages.

**Conclusion:**

We identified strategies to characterize the diversity of the MS population in Canada.

## 1 Introduction

Multiple sclerosis (MS) is heterogeneous regarding presentation and outcomes. A growing body of evidence indicates that demographic and diversity-related characteristics (hereinafter ‘diversity’) of the individual and of their socio-political environment influence outcomes in many health conditions, including MS (1–3). Diversity encompasses factors such as sex, gender, sexual orientation, age, race, ethnicity, comorbidity and social determinants of health. To develop approaches to ensuring equitable health care and optimal outcomes for everyone with MS in Canada, the diversity of the MS population must be known. However, our prior scoping review found that we lack a clear understanding of the diversity of the MS population (4).

Potential data sources that capture information about people with MS include administrative databases, surveys, and medical records, each with strengths and limitations, including variable capture of diversity factors. Thus, multiple approaches may be needed to fully characterize the population, but these vary in feasibility and utility. We aimed to identify promising strategies to fill information gaps regarding diversity characteristics of the MS population in Canada using a nominal group technique (NGT). NGT are intended for use by small groups to reach consensus on identifying key problems or developing solutions to complex problems (5).

## 2 Methods

We obtained ethics approval from the Nova Scotia Health Research Ethics Board. Participants external to the research team provided e-consent via REDCap (6) before participation.

### 2.1 Facilitation

To enhance geographic diversity and to limit costs and carbon footprint, we conducted the NGT process using a virtual platform (5), Microsoft Teams, hosted by Nova Scotia Health. Since the COVID-19 pandemic an increasing number of investigators are using virtual adaptations of the NGT (7). After introductions, the facilitator (RAM) clarified that the meeting objective was to address the question: *What strategy would best address the information gaps regarding diversity of the MS population in Canada?* and summarized the NGT process. The facilitator discussed the findings of prior scoping review which found that the most common characteristics reported were age and sex, and that most characteristics of interest (such as sexual orientation, race, ethnicity, religion) were rarely or never reported (4).

### 2.2 Participants and sample size

The group included the study investigators, who had expertise in MS, epidemiology, biostatistics, qualitative methods, primary data collection and secondary data analysis. The investigator group was expanded to include three additional participants with expertise in MS and/or equity, diversity and inclusion. Collectively, the group included investigators who were early, mid and late career researchers, from the eastern and western halves of Canada, with varying racial and ethnic backgrounds. Most NGT groups involve 6–8 participants although they are occasionally slightly larger; large groups make the NGT process (7, 8) inefficient and generate too many ideas.

### 2.3 Silent idea generation

Participants had 10 minutes for silent idea generation with cameras and microphones off to allow time for thought and reflection and to limit pressure to choose one idea prematurely.

### 2.4 Round robin recording of ideas

At the end of the silent idea generation step, participants turned on their cameras. Each participant provided one idea, which was transcribed on screen. Once each participant had contributed one idea, the facilitator restarted the process until all participants had no further ideas to contribute. This process promotes engagement by everyone, allows conflicting ideas to emerge, and aids management of many ideas. The facilitator reminded participants of the types of ideas of interest as needed.

### 2.5 Serial discussion

After all ideas were shared, each idea was discussed to clarify it, and delineate strengths, limitations, and areas of disagreement.

### 2.6 Preliminary voting

In this phase, participants rated ideas to reduce the list of ideas. Participants anonymously scored each idea with respect to: utility and feasibility, each on a 7-point numeric scale ranging from not useful/not feasible to extremely useful/feasible [not, slightly, somewhat, moderately, fairly, very, extremely]. Seven point numeric scales are commonly used to assess usability and feasibility, and in consensus-based studies (9, 10). We used the Microsoft Forms app (via Microsoft Teams) to capture the scores. The forms app was pilot-tested using dummy ideas before the meeting.

### 2.7 Discussion of preliminary voting

The group reviewed the outcome of the voting and identified ideas where voting patterns were inconsistent or had less support. Most ideas moved forward for final voting. Similar ideas were combined into one idea.

### 2.8 Final voting

The anonymous final vote ranked the ideas to prioritize them using Microsoft Forms. This provided the meeting outcome and closed the process.

The NGT process took 105 min.

## 3 Results

Eight people attended the NGT meeting including the facilitator. Ten general strategies were proposed that were directly related to the meeting objective (Table 1). Following the initial voting, adopting a standardized clinic intake form for use in MS Clinics across Canada had the highest observed median usefulness score (very) (Table 1). Use of novel linkages of existing data and novel applications of administrative data were perceived to be the next most useful strategies (fairly useful); these strategies were recognized as having some overlap. A national survey, collecting the data through MS Canada interactions, and use of the Canadian Primary Care Sentinel Surveillance Network data received the lowest usefulness ratings (somewhat).

**Table 1:** Potential strategies to fill information gaps regarding diversity of the MS population in Canada.

Idea (strategy)	Usefulness Median (p25, p75)	Feasible Median (p25, p75)
Standardized form capturing diversity characteristics for electronic health records across Canada/Consent everyone attending an MS Clinic at initial visit to complete a standardized form	Very (fairly, very)	Fairly (moderately, very)
Develop new ways/applications of administrative data to understand diversity	Fairly (moderately, very)	Fairly (fairly, fairly)
Use novel data linkages (e.g., IRCC, inter-RAI, CCHS, CLSA, CANPATH)	Fairly (fairly, very)	Fairly (fairly, very)
Restart the Canadian MS Monitoring System Initiative	Moderately (somewhat, fairly)	Moderately (slightly, moderately)
Mine electronic health records using artificial intelligence	Moderately (somewhat, fairly)	Somewhat (slightly-somewhat, moderately)
Advocacy for enhanced data collection & reporting through federal institutions/structures: CCHS to capture MS, CIHI to mandate enhanced data capture & reporting	Moderately (somewhat, moderate)	Slightly (slightly, slightly-moderately)
Use Canadian Primary Care Sentinel Surveillance Network	Somewhat (somewhat, fairly)	Somewhat (somewhat, fairly)
Survey people with MS nationally	Somewhat (somewhat, moderately)	Moderately (somewhat, moderately)
MS Canada collects diversity information for everyone who contacts them through the Navigator program	Somewhat (slightly, moderately)	Moderately (somewhat, fairly)

CANPATH, Canadian Partnership for Tomorrow Project; CIHI, Canadian Institute for Health Information; CCHS, Canadian Community Health Survey; CLSA, Canadian Longitudinal Study on Aging; IRCC, Immigration, Refugees and Citizenship Canada; p25, 25th percentile; p75, 75th percentile.

Feasibility and usefulness ratings differed slightly. Although a standardized clinic form attained the highest feasibility rating reported, this was slightly lower (fairly) than its usefulness and thus was the same as for the use of novel data linkages and novel applications of administrative data. The lowest ranked strategy for feasibility were advocacy for enhanced data collection and reporting through federal institutions and structures such as the Canadian Institutes for Health Information, mining electronic health records using artificial intelligence, using the Canadian Primary Care Sentinel Surveillance System, and collecting data through MS Canada.

**Figure 1:**
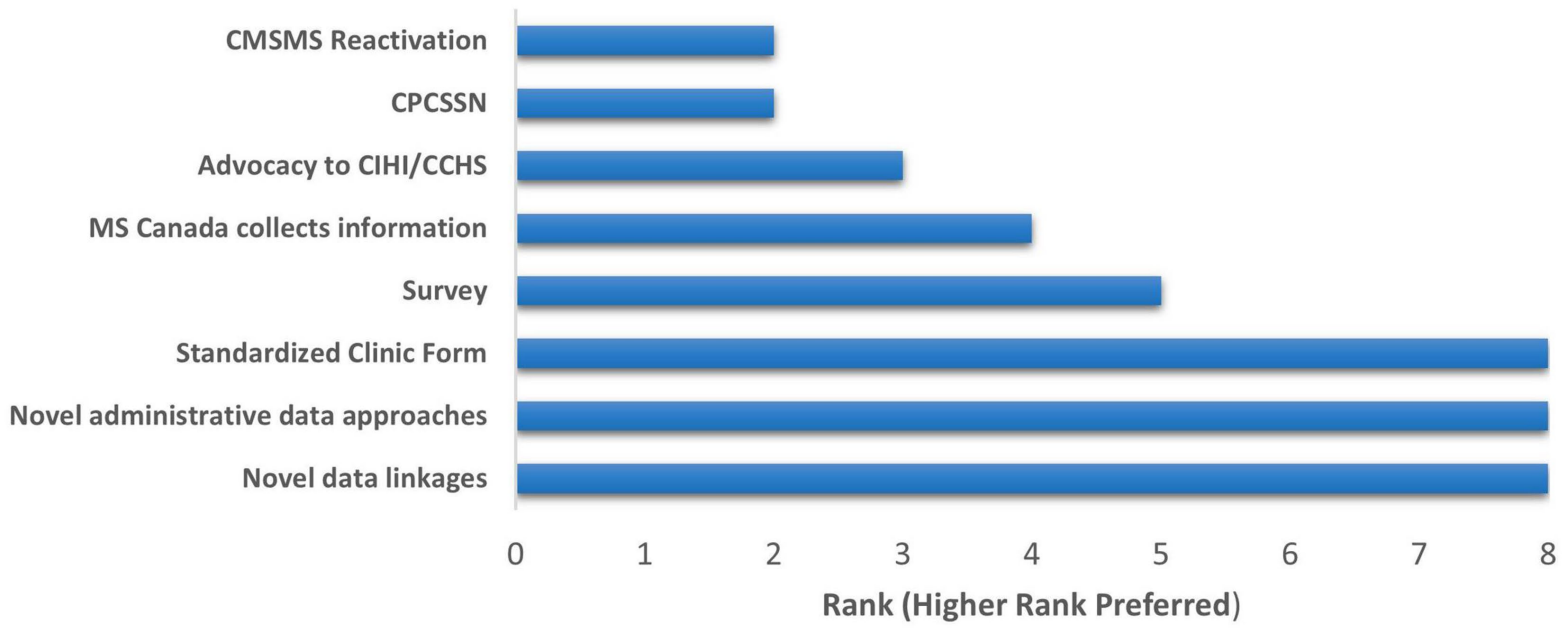
Ranking of strategies for characterizing the diversity of the multiple sclerosis population in Canada. CMSMS, Canadian Multiple Sclerosis Monitoring System Initiative; CPCCSN, Canadian Primary Care; Sentinel Surveillance Network; CIHI, Canadian Institute of Health Information; CCHS, Canadian Community Health Survey; MS, multiple sclerosis.

The strategies, including strengths, limitations (Supplementary Table 1), and ratings were discussed; artificial intelligence was dropped from final voting. Following final voting to rank the remaining eight options, the top three options were tied: use of a standardized clinic form nationally, novel applications of administrative data, and novel data linkages (Figure 1). Use of a national survey was ranked fourth. Using the Canadian Primary Care Sentinel Surveillance Network (an electronic medical record-derived primary care database) and reactivating the Canadian MS Monitoring System Initiative (a defunct surveillance initiative to improve care) were ranked lowest.

## 4 Discussion

We used a structured process to reach consensus to limit problems related to group dynamics and social influences and included participants from across Canada with different disciplinary and research backgrounds. We identified several strategies with varying usability and feasibility, the most popular of which were use of a standardized clinic form nationally, novel applications of administrative data, novel data linkages, followed by a national survey.

The adoption of standardized data collection form across Canadian MS Clinics was viewed as feasible given the increasing adoption of electronic health records across Canada; many of the electronic health records include portals for direct data collection from patients. While most people with MS regularly access care, some people with MS do not attend MS Clinics, precluding complete capture of the population. A standardized form would require consensus across more than 25 clinics. Changes in existing forms or templates in electronic health records can also be costly. A prior effort by the Canadian Institute of Health Information to develop and capture a voluntary minimum dataset from MS Clinics across Canada, the Canadian Multiple Sclerosis Monitoring System, was discontinued after 4 years due to poor uptake, although this preceded adoption of electronic health records in most MS Clinics. Interoperability issues also exist given the use of many different electronic health records, although increasing adoption of the Fast Healthcare Interoperability Resources (FHIR) (11) standard should mitigate some of these issues.

Administrative (health claims) data are an accessible and cost-efficient data source compared with primary data collection, and in Canada they are population-based. They are already used for chronic disease surveillance as part of the Canadian Chronic Disease Surveillance System (12), including for MS and are well-suited to repeated use over time. They are recognized to be of high quality, and have been used to address a wide range of research questions related to MS. However, there are several challenges related to using these data. First, Canada has 10 provinces and 3 territories, each of which collects administrative data using its own systems and does not permit the data to leave the province of origin. Thus, researchers must pursue different data access approval processes in multiple regions, deal with potential differences in data organization and comparability, and use federated approaches for data analysis, increasing time required, costs, and complexity (13). Efforts are ongoing to support researchers in addressing these challenges of multijurisdictional research, including the development of the Health Data Research Network (HDRN) Canada (13). The HDRN includes a data access support hub which summarizes key data access requirements, provides resources regarding privacy and data sovereignty, and datasets, and algorithms (such as to identify MS) that have been validated or feasibility-tested. Second, these data sources largely lack information about race or ethnicity, gender identity, sexual orientation, and key social determinants of health. However, some provinces, such as Manitoba and Nova Scotia, have initiated programs to capture race and ethnicity at the time of registration for universal health care (14). This has been supported by the Canadian Institute for Health Information which has released Canadian standards for collection of race-based and Indigenous identity data (15). Moreover, emerging methods may allow identification of people who are transgender (16), and area-level deprivation measures can be derived by linkage of residential postal code to Census Data. Future work should examine ways to identify other diversity characteristics using administrative data. Another option is linkage of multiple novel data sources may provide a means to provide missing information. For example, the Immigration Refugees and Citizenship Canada captures information related to race, ethnicity, education, and country of birth for immigrants and refugees. Although availability varies by province or territory, some jurisdictions can link in datasets regarding use of social housing, employment/income assistance, contact with the justice system, and educational performance (17). Existing provincial and national surveys and cohort studies can also be linked to administrative data (18). However, linkage of multiple datasets is complex, requires engaging multiple data custodians, and may mean that the final linked dataset is no longer population-based. As awareness of the importance of addressing health inequities grows, these processes and population coverage may improve.

Standalone national surveys are more likely to capture information that is missing from administrative data sources, but would likely be limited to the community-dwelling population (and generally healthier participants) (19), excluding the ~5% of people with MS residing in long-term care (20). Such surveys are easy to develop and relatively low cost, and do not require the approval of multiple data custodians; however, response rates to medical surveys are low and declining (21–23). Moreover, responses may differ according to diversity characteristics (23). Repeated surveys would be needed to evaluate changes in diversity characteristics of the MS population over time, amplifying costs.

While we focused on Canada, these strategies could be used in other jurisdictions. Some countries, such as France and Sweden for example, have developed national registries which use standardized data collection regarding people with MS. The presence of existing registries would facilitate capturing additional information about diversity characteristics within the registry or via data linkage (24, 25). Many countries with universal, public health insurance have administrative data sources (see Appendix-e4 of (26) for an inventory of these data sources from many world regions, including data access procedures), which have been used to address various MS-related research questions (27). Some of these data sources suffer from similar gaps in diversity information as do administrative data in Canada (28), and data linkage strategies have similarly been proposed to mitigate these gaps (25).

The size of our nominal group was dictated by recommendations for NGT processes because larger groups can fail to reach consensus, but we acknowledge that use of multiple groups is possible, and that choice of participants can also influence the findings. A scoping review suggested that recruitment of participants with experience related to the NGT topic is more likely to produce items that are directly relevant, increases face validity, and is likely to produce an adequate range of items. However, it is possible that including participants with a broader range of experiences, including people with MS or caregivers could have provided additional insights and more generalizable items. Including different interest holders in a single group can make it more challenging to reach consensus and potentially introduce power differentials. Use of multiple nominal groups involving different groups of interest holders could address this issue, but take more time, and analysis may be more complex due to use of different terms for similar items, or different interpretations of items with the same names. The time constraints of the nominal group process can limit the opportunity for deep exploration of each idea. However, the time frame is important because the format is highly structured and requires sustained concentration throughout the process. Longer time frames risk cognitive fatigue, loss of engagement, and drift from the structured process. Practically, it can often be difficult to engage clinician-investigators and investigators in lengthy meetings due to their limited availability. Finally, the findings reflect opinions at a discrete point in time.

We identified multiple strategies to fill information gaps regarding diversity characteristics of the MS population in Canada. These included use of standardized forms across MS Clinics, novel data linkages, and novel applications of administrative data (which could also be included in data linkages), and a national survey. Each of these approaches has strengths and limitations. Overall, multiple data sources and approaches will likely be needed to fully characterize the MS population, but these potentially vary in feasibility and utility. These strategies provide a starting point for future work including detailed elucidation of implementation considerations.

## Data Availability

The original contributions presented in the study are included in the article/Supplementary material, further inquiries can be directed to the corresponding author.
