# Supplementary material for: Identifying strategies to characterize the diversity of the MS population in Canada: a nominal group study
Source: Front Neurol. 2026 Mar 31;17:1757659. doi: 10.3389/fneur.2026.1757659 (PMC13076133; doi:10.3389/fneur.2026.1757659)
Supplement: Supplementary file 1 [file Table_1.DOCX]

Supplementary Material

# Supplementary Data

Table e1. Overview of potential strategies

| **Strategy** | **Strengths** | **Weaknesses** | **Opportunities** |
| --- | --- | --- | --- |
| Standardized form in clinic | - Most people with MS access health care regularly - MS Clinic is a common source of care - Some clinics already collect some of this information | - Requires consensus on content across >25 MS Clinics across Canada; local requirements may limit ability to reach consensus - Differing data systems across clinics and most do not have research databases (or staff to support clinical databases) - Some people with MS do not attend MS Clinics thus may not be generalizable - Costs of implementation may be substantial | - Increasing use of electronic health records, patient portals across Canada ease implementation |
| Use Canadian Primary Care Sentinel Surveillance Network | - Captures individuals seeking primary care - Validated algorithm exists for identifying people with MS | - Captures ~3,000 people with MS, representing less than 3% of the MS population in Canada - Other than age, sex, region, chronic conditions, diversity characteristics are not captured | - Can be linked to administrative datasets - In future, may be possible to link to other datasets |
| Novel data linkages (e.g. IRCC, inter-RAI, CCHS, CLSA, CANPATH) | - Multiple datasets that each capture some of the diversity characteristics of interest and could be linked to provide more comprehensive picture of MS population | - Once multiple datasets are linked, no longer population-based because whole population is not represented in each dataset, reducing generalizability - Linkages may be needed in multiple regions, further adding to complexity - Potential need for approval from multiple data custodians - Data may not be collected for research so may need validation | - Some datasets are population-based - Growing number of datasets with diversity information available for linkage |
| Administrative data | - Population-based in all provinces and territories - Emerging methods to identify individuals who are transgender^1^ - Moderate cost - Repeatable | - Data cannot leave the province/territory of origin, so each regional dataset must be analyzed separately (although this has been accomplished for the Canadian Chronic Disease Surveillance System) - Capture predominantly age, sex, region of residence - Not collected for research, so need to be validated - Historically have not captured race, ethnicity, religion, sexual orientation, gender identity, and so on. - Linkage to other datasets can result in loss of population-based nature of the data | - Some provinces/territories beginning to capture race at time of health care card renewal/ hospitalization - CIHI has developed data standards for race, gender identity to support adoption - Some limitations can be addressed by data linkage |
| Survey people with MS nationally | - Low cost - Straightforward to develop - Electronic surveys are easy to conduct - Can capture detailed diversity information - Repeatable | - Low and declining response rates to medical surveys reduce generalizability - Electronic surveys will miss individuals without broadband access - Repeated surveys may not be linkable | - To align with data collection of other national initiatives |
| MS Canada collects diversity information for everyone who contacts them through the Navigator program | - Under direct purview of MS Canada, making data accessible for MS Canada programs/ policies/advocacy | - Some people with MS not connected with MS Canada - Providing information may raise concerns regarding discrimination/act as a barrier to services - Need infrastructure to collect information in standardized manner | - Align data collection with other national initiatives to allow data harmonization & comparisons |
| Restart the Canadian MS Monitoring System Initiative | - CIHI previously developed infrastructure - Successful pilot tests by three clinics some of whom have continued data collection - Linkable to other CIHI data holdings | - Costly for clinics, provinces/territories and CIHI - Previously failed due to insufficient engagement by provincial health authorities |  |
| Mine electronic health records using artificial intelligence | - Large language models are being developed to identify diversity characteristics^2^ allowing data extraction even if it has not been entered into structured fields. | - Even in one province, there may be multiple electronic health records with many different data custodians - Models developed in one health record/population/region may not “transfer” well to others - AI may be prone to bias, including related to diversity characteristics - Depend on the accuracy of the information in the electronic health record, and may capture information from the perspective of the provider rather than the patient - Information must be captured in the health record for it to be identified | - Most provinces have or are adopting electronic health records - AI continuing to develop and increasingly adopted for tasks within health systems (such as transcription) |
| Advocacy for enhanced data collection & reporting through federal institutions/ structures: CCHS to capture MS, CIHI to mandate enhanced data capture & reporting | - Integration of information about MS into existing data capture systems enhances feasibility, reducing costs - CIHI sets data standards for data it receives from the provinces/territories | - Although new data standards regarding race, ethnicity and gender identity have been proposed by CIHI, most administrative data in Canada still lack this information | - CCHS is distributed annually, captures multiple health determinants, and is representative of the population in Canada - Could advocate with other health charities as was successfully done for the National Population Health Study of Neurological Disease previously^3^ |

Abbreviations: CANPATH = Canadian Partnership for Tomorrow Project, CCHS = Canadian Community Health Survey, CIHI = Canadian Institute for Health Information, CLSA = Canadian Longitudinal Study on Aging, IRCC = Immigration, Refugees and Citizenship Canada

Canadian Primary Care Sentinel Surveillance System: collects de-identified information from electronic medical records of participating primary care practices across Canada.^4^ Algorithm to identify MS has been validated.^5^

Canadian MS Monitoring System Initiative: initiative developed with the goal of improving care and quality of life of people with MS in Canada by measuring disease patterns, identifying variation in treatment and care between people and across time, and monitoring long-term outcomes. A pilot phase was completed successfully, but the system was not adopted across Canada thus the initiative was discontinued.

CIHI: independent, not-for-profit, corporation that collects, maintains and integrates health information to support health system management and policy decisions in Canada

MS Canada Navigator: Navigators are part of MS Canada’s knowledge network, which provides information and support to people with MS across Canada

**References**

1. Rytz CL, King JA, Saad N, et al. Validating a Case Definition for Transgender Adults Using Administrative Data. JAMA Netw Open 2025;8:e2451700.

2. Guevara M, Chen S, Thomas S, et al. Large language models to identify social determinants of health in electronic health records. npj Digital Medicine 2024;7:6.

3. Neurological Health Charities Canada, The Public Health Agency of Canada, Health Canada, The Canadian Institutes of Health Research. Mapping connections: An understanding of neurological conditions in Canada. Ottawa, ON: Public Health Agency of Canada, 2014 September 2014.

4. Williamson T, Green ME, Birtwhistle R, et al. Validating the 8 CPCSSN case definitions for chronic disease surveillance in a primary care database of electronic health records. Ann Fam Med 2014;12:367-372.

5. Marrie RA, Kosowan L, Taylor C, Singer A. Identifying people with multiple sclerosis in the Canadian Primary Care Sentinel Surveillance Network. Multiple Sclerosis Journal - Experimental, Translational and Clinical 2019;5:2055217319894360.

**
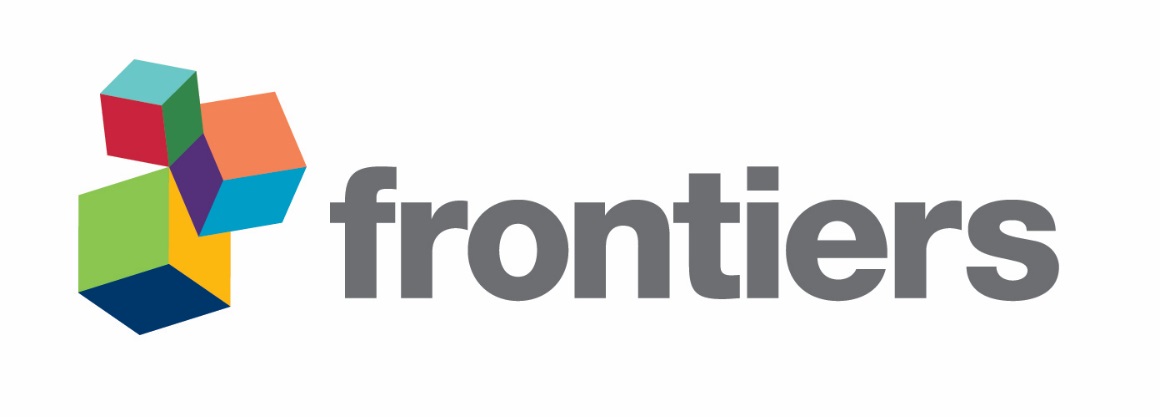
**
